# Aseptic thoracic spondylodiscitis (pseudo discitis) in a septuagenarian without predisposing factors

**DOI:** 10.1016/j.clinsp.2024.100571

**Published:** 2025-01-24

**Authors:** Ana C. Fiorini, Carla Alexandra Scorza, Fulvio Alexandre Scorza, Josef Finsterer

**Affiliations:** a Programa de Estudos Pós-Graduado em Fonoaudiologia, Pontifícia Universidade Católica de São Paulo (PUC-SP), São Paulo, SP, Brazil; b Departamento de Fonoaudiologia, Escola Paulista de Medicina, Universidade Federal de São Paulo (EPM/UNIFESP), São Paulo, SP, Brazil; c Escola Paulista de Medicina, Universidade Federal de São Paulo (EPM/UNIFESP), São Paulo, SP, Brazil; d Neurology Neurophysiology Centre, Vienna, Austria

Spondylodiscitis (SD) is usually an infectious inflammation of one or more vertebral bodies (osteomyelitis, spondylitis), the intervertebral disc (discitis) and the paravertebral soft tissue.^1^ Etiologically, SD can be caused pyogenically by bacteria, granulomatously by tuberculosis or fungi, or by parasites (e.g., echinococci).^1^ The most common infectious agents are Staphylococcus aureus (90 % of cases) and streptococci.^2^ In the majority of cases, the pathogens reach the anterior vertebral bodies through hematogenous spread.^2^ More rarely, spread occurs per continuitatem (e.g., from a paravertebral abscess) or by direct inoculation through surgery, lumbar puncture or trauma.^2^ Risk factors for the development of SD include advanced age, diabetes, sepsis, intravenous drug abuse, intravenous line contamination, urinary tract infections, immunodeficiency, previous spinal surgery or trauma.^3^ The prevalence of SD is estimated at 5–6/100,000/a.^2^

Aseptic spondylodiscitis (ASD) is an even rarer disease characterized by radiological and biopsy evidence of spondylodiscitis in the absence of an infectious agent in any compartment. ASD has been reported in HLAb27-positive ankylosing spondylitis,^4^ after spinal surgery (e.g., microdiscectomy, chemonucleolysis),^5^ after repair of an aortic aneurysm,^6^ polymyalgia rheumatica,^7^ calcific nucleopathy, Behcet's disease,^8^ lumbar puncture^9^ and in synovitis, acne, pustulosis, hyperostosis and aseptic osteitis (SAPHO) syndrome.^10^ An aging male with ASD but without any of the risk factors for SD and without conditions previously reported in association with ASD has not been described.

The patient is a 76-year-old man, 180 cm tall, weighing 117 kg, who was admitted because of pain in the lower thoracic spine with girdle-shaped radiation and paresthesias in both feet. The medical history was otherwise positive for arterial hypertension, hypothyroidism, hyperlipidemia, umbilical hernia, sigmoid diverticulosis, prostatic hyperplasia, overflow bladder, bladder diverticulum, hydronephrosis-2, previous renal failure, previous renal pelvisplasty, left total knee arthroplasty, and multisegmental spinal degeneration with disc protrusion, high-grade foraminal stenosis, and mild vertebral stenosis. The medical history was negative for spinal trauma, fever, infection, stroke or hemorrhage shortly before the onset of back pain. Due to prostatic hyperplasia and urinary dysfunction, he received an indwelling catheter shortly after admission and later a suprapubic catheter, which was complicated by hematomas between the base of the bladder and the prostate and in the abdominal wall. His current medication includes carvedilol, amlodipine, doxazosin, valsartan, L-thyroxine, dutasteride, hydromorphone, paracetamol, pantoprazole and macrogol.

The clinical neurological examination two days before the operation revealed only a postural tremor of the hands and bilateral leg edema. Repeated blood tests during the hospital stay revealed only mild anemia, a slightly elevated CRP after surgery and occasional mild renal insufficiency. MRI of the thoracic spine revealed clear signs of spondylodiscitis Th9/10 with contrast enhancement of both vertebrae, deformity of the Th9/10 disc and an epidural abscess at this level (Fig. 1). The patient underwent successive dorsal spondylodesis Th7-Th12, interarcuate decompression, drainage of the abscess and antibiotic therapy with cefazolin. After a biopsy of the affected vertebra, culture of the abscess, and repeated blood cultures (6 times), no infectious agent could be detected. The erythrocyte sedimentation rate and HbA1c level were normal, and the quantiferon test, HLAb27, and rheumatoid factor were negative. The only possible portal of entry for SD was nail bed suppuration, which was resolved after local therapy. After surgery and rehabilitation, the patient recovered almost completely within two months, especially with regard to pain and sensory disturbances.

**Fig. 1:**
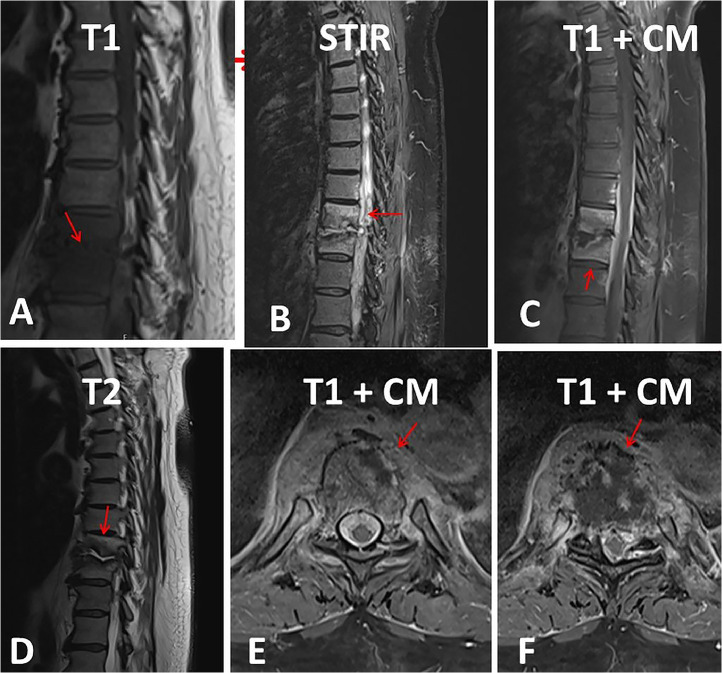
MRI of the spine with pronounced spondylodiscitis. The Th9/10 T2-weighted disc shows high signal intensity and is significantly narrowed (panels B and C). The base plate of the BWK 9 and the cover plate of the Th10 are irregular and show pronounced signal changes; There is a reduction in the height of both vertebral bodies, with the depression in the base plate of Th9 being emphasized on the right and the depression in the cover plate of BWK 10 being emphasized on the left (panels A, B, C, D). In the STIR sequence there are hyperintense bone marrow signal changes, which are shown with a low signal on T1 (panel B). After contrast, significant enhancement of Th9 and Th10 and the surrounding soft tissues (panels C, E, F). At the Th9/10 level, fusiform contrast enhancement dorsal to the vertebral bodies in the spinal canal over a longitudinal extent of 7.8 cm, with a width of up to 6 mm, corresponding to an epidural abscess, with consecutive constriction of the dural sac (panel B). The myelon itself shows no signal change.

The index patient is of interest because he has an ASD involving the Th9/Th10 vertebra without classic risk factors for SD and because his medical history does not reveal any diseases previously associated with an ASD. The medical history, clinical presentation, blood tests, MRI of the spine and the positive effect of the surgery all speak in favor of ASD in the index patient. The cause of the ASD remains unknown, but it can be speculated that a causative infectious agent did not grow on the blood cultures or that the subvesical hematoma had an immunosuppressive effect. The elevated CRP was attributed to the subvesical hemorrhage or the surgery. ASD after laser disc decompression was attributed to heat exposure.^11^

The differential diagnoses that were excluded in the index patient included activated or erosive osteochondrosis, tuberculous spondylitis (> 50 % height reduction, abscess of a vertebra with peripheral enhancement, involvement of two vertebrae, paravertebral abscesses with calcifications in half of the cases, destruction of the pedicles in half of the cases), pathological fractures, primary spinal malignancies, metastases, ankylosing spondylitis, Scheuermann's disease, hemodialysis-associated spondyloarthropathy, rheumatoid arthritis and SAPHO syndrome.^4^, ^5^, ^6^, ^7^, ^8^, ^9^, ^10^ A limitation of the study is that the patient was not tested for SARS-CoV-2, which has been reported in association with SD.^12^

The MRI was interpreted as SD based on the typical radiological features. These include erosions of the T1 and T2 hypointense endplates and severe vertebral body edema with relatively flat, homogeneous contrast uptake, which usually extends at least in places through the entire vertebral body to the opposite endplate. If the intervertebral disc is affected, a T1-hypointense and T2-/STIR-hyperintense abnormal signal is found. In the early stages of SD, there may be band-like contrast enhancement close to the endplate, which later directly transitions into enhancement of the vertebral bodies. Over time, there is a reduction in the height of the disc space and disc herniation. Areas with purulent fusion in the vertebral body or the intervertebral disc show a diffusion restriction. In the paravertebral spread, T2 hyperintense soft tissue edema and diffuse or marginal contrast uptake are seen, indicating epidural spread (phlegmon or meningeal abscess), with T2 hyperintense abscess contents. In individual cases, there may also be circumscribed involvement of a facet joint.

In summary, this case shows that ASD can occur even when no disease associated with ASD is present. In patients with back pain, ASD should be suspected if there are no classic risk factors for SD, blood tests are near normal, and there are no conditions previously reported in association with ASD. Surgical treatment of ASD can lead to almost complete resolution of symptoms.

## Ethical approval

Has been provided by the institutional review board.

## Consent to participation

Not applicable.

## Consent for publication

The patient consented with the publication.

## Funding

None received.

Availability of data and material: all data are available from the corresponding author.

## Authors’ contributions

JF was responsible for the design and conception, discussed available data with coauthors, wrote the first draft, and gave final approval. CS, FS, and AF: contributed to literature search, discussion, correction, and final approval.

## Conflicts of interests

The authors declare that the research was conducted in the absence of any commercial or financial relationships that could be construed as a potential conflict of interest.
